# Optimizing warfarin dosing for patients with atrial fibrillation using machine learning

**DOI:** 10.1038/s41598-024-55110-9

**Published:** 2024-02-24

**Authors:** Jeremy Petch, Walter Nelson, Mary Wu, Marzyeh Ghassemi, Alexander Benz, Mehdi Fatemi, Shuang Di, Anthony Carnicelli, Christopher Granger, Robert Giugliano, Hwanhee Hong, Manesh Patel, Lars Wallentin, John Eikelboom, Stuart J. Connolly

**Affiliations:** 1 Centre for Data Science and Digital Health, Hamilton Health Sciences, Hamilton, ON Canada; 2 Population Health Research Institute, Hamilton, ON Canada; 3 Division of Cardiology, Department of Medicine, McMaster University, Hamilton, ON Canada; 4 Institute of Health Policy, Management and Evaluation, University of Toronto, Toronto, ON Canada; 5 Department of Statistical Sciences, University of Toronto, Toronto, ON Canada; 6 Department of Computer Science, University of Toronto, Toronto, ON Canada; 7 Department of Electrical Engineering and Computer Science, Massachusetts Institute of Technology, Cambridge, MA USA; 8 Institute for Medical and Evaluative Sciences, Massachusetts Institute of Technology, Cambridge, MA USA; 9 Vector Institute, Toronto, ON Canada; 10 Department of Cardiology, University Medical Center, Johannes Gutenberg University Mainz, Mainz, Germany; 11 Microsoft Research, Montreal, QC Canada; 12 Dalla Lana School of Public Health, University of Toronto, Toronto, ON Canada; 13 Division of Cardiology, Department of Medicine, Duke University Medical Center, Durham, NC USA; 14 Duke Clinical Research Institute, Duke University, Durham, NC USA; 15 Division of Cardiovascular Medicine, Brigham and Women’s Hospital, Harvard Medical School, Boston, MA USA; 16 Department of Biostatistics and Bioinformatics, Duke University School of Medicine, Durham, NC USA; 17 Department of Medical Sciences, Cardiology, Uppsala University, Uppsala, Sweden; 18 Uppsala Clinical Research Center, Uppsala University, Uppsala, Sweden; 19 Division of Hematology and Thromboembolism, Department of Medicine, McMaster University, Hamilton, ON Canada

**Keywords:** Atrial fibrillation, Machine learning

## Abstract

While novel oral anticoagulants are increasingly used to reduce risk of stroke in patients with atrial fibrillation, vitamin K antagonists such as warfarin continue to be used extensively for stroke prevention across the world. While effective in reducing the risk of strokes, the complex pharmacodynamics of warfarin make it difficult to use clinically, with many patients experiencing under- and/or over- anticoagulation. In this study we employed a novel implementation of deep reinforcement learning to provide clinical decision support to optimize time in therapeutic International Normalized Ratio (INR) range. We used a novel semi-Markov decision process formulation of the Batch-Constrained deep Q-learning algorithm to develop a reinforcement learning model to dynamically recommend optimal warfarin dosing to achieve INR of 2.0–3.0 for patients with atrial fibrillation. The model was developed using data from 22,502 patients in the warfarin treated groups of the pivotal randomized clinical trials of edoxaban (ENGAGE AF-TIMI 48), apixaban (ARISTOTLE) and rivaroxaban (ROCKET AF). The model was externally validated on data from 5730 warfarin-treated patients in a fourth trial of dabigatran (RE-LY) using multilevel regression models to estimate the relationship between center-level algorithm consistent dosing, time in therapeutic INR range (TTR), and a composite clinical outcome of stroke, systemic embolism or major hemorrhage. External validation showed a positive association between center-level algorithm-consistent dosing and TTR (R^2^ = 0.56). Each 10% increase in algorithm-consistent dosing at the center level independently predicted a 6.78% improvement in TTR (95% CI 6.29, 7.28; *p* < 0.001) and a 11% decrease in the composite clinical outcome (HR 0.89; 95% CI 0.81, 1.00; *p* = 0.015). These results were comparable to those of a rules-based clinical algorithm used for benchmarking, for which each 10% increase in algorithm-consistent dosing independently predicted a 6.10% increase in TTR (95% CI 5.67, 6.54, *p* < 0.001) and a 10% decrease in the composite outcome (HR 0.90; 95% CI 0.83, 0.98, *p* = 0.018). Our findings suggest that a deep reinforcement learning algorithm can optimize time in therapeutic range for patients taking warfarin. A digital clinical decision support system to promote algorithm-consistent warfarin dosing could optimize time in therapeutic range and improve clinical outcomes in atrial fibrillation globally.

Stroke killed an estimated 6,190,000 people in 2019, making it the 2nd leading cause of death worldwide^1^. A leading cause of stroke is atrial fibrillation, a heart arrythmia that imposes a five-fold increase in the risk of ischemic stroke^2^. While novel oral anticoagulants are increasingly used to reduce risk of stroke in patients with atrial fibrillation, vitamin K antagonists such as warfarin continue to be commonly used for stroke prevention across the world^3,4^, and remains the only oral option for patients with conditions such as mechanical heart valves.

While effective in reducing the risk of stroke, vitamin K antagonists like warfarin are difficult to use clinically. They interact with a range of foods and drugs^5^, and thus require ongoing laboratory monitoring and frequent dose adjustments to maintain adequate anticoagulation. In clinical practice, this is accomplished by monitoring plasma International Normalized Ratio (INR), which evaluates coagulation. The established therapeutic range for atrial fibrillation is an INR of 2.0–3.0, and extended time in therapeutic range (TTR) is associated with a lower risk of stroke^6^. However, given the complex pharmacodynamics of warfarin, achieving a high TTR can be difficult and thus many patients on warfarin experience inadequate anticoagulation^7^.

Clinical and computerized algorithms have been developed to support clinicians in addressing the challenges of managing warfarin, and both have been shown to improve TTR^8–12^. While effective, most of these algorithms are relatively simple, typically providing clear decision rules based on INR thresholds (such as increasing the dose of warfarin by 10% if INR = 1.5–2.0). This approach has the advantage of being highly interpretable and easy to implement, but the potential disadvantage of being ‘one-size fits all’, in that the same set of rules are applied irrespective of a patient’s age, ethnicity, concurrent medications, and other factors that may be associated with warfarin metabolism. Indeed, there is evidence that more data-driven approaches to warfarin dosing could provide incremental gains in TTR over existing algorithms, further reducing the risk of stroke in patients with atrial fibrillation^11,12^.

Warfarin dosing for atrial fibrillation can be framed as a dynamic treatment regime problem^13^. Dynamic treatment regimes are sets of rules or policies that describe how treatments are to be assigned in response to a patient’s state, including dynamically changing factors like INR^14,15^. Initial approaches to optimizing dynamic treatment regimes employed statistical methods^16^, but there is growing interest in reinforcement learning (RL) for this application^17,18^. RL is a branch of machine learning in which an agent learns an optimal policy through interactions with an environment, aiming to maximize a reward function. RL has been applied with varying levels of success to dynamic treatment regime problems in sepsis^19^, cancer^20^, epilepsy^21^, diabetes^22^, schizophrenia^23^, anemia^24^, and HIV^25^.

We used a novel semi-Markov decision process^26^ formulation of the Batch-Constrained deep Q-learning^27^ algorithm to develop an RL model that can dynamically recommend optimal warfarin dosing for patients with atrial fibrillation (Fig. 1). We developed and evaluated this model on four large non-overlapping data sets made up of patients in the control groups of the pivotal randomized clinical trials of dabigatran (RE-LY)^28^, edoxaban (ENGAGE AF-TIMI 48)^29^, apixaban (ARISTOTLE)^30^ and rivaroxaban (ROCKET AF)^31^, made available to the research team as part of the COMBINE AF collaborative^32^. Each of these clinical trials compared a different anticoagulant to warfarin, providing us with a total sample (following data cleaning) of 28,232 patients on warfarin with INR monitoring, followed for a median of 22.2 months, for a total of 892,333 dose–response pairs for training and evaluation. The use of randomized control trial data for reinforcement learning model development is novel and provides several notable advantages over most routinely collected data sets, including dedicated data collection and on-site monitoring to ensure high data quality, a clinically representative set of baseline patient features for accurate modeling of the state space, a more statistically robust approach to evaluation, and more generalizability thanks to a study population that includes patients living across six continents.

**Figure 1:**
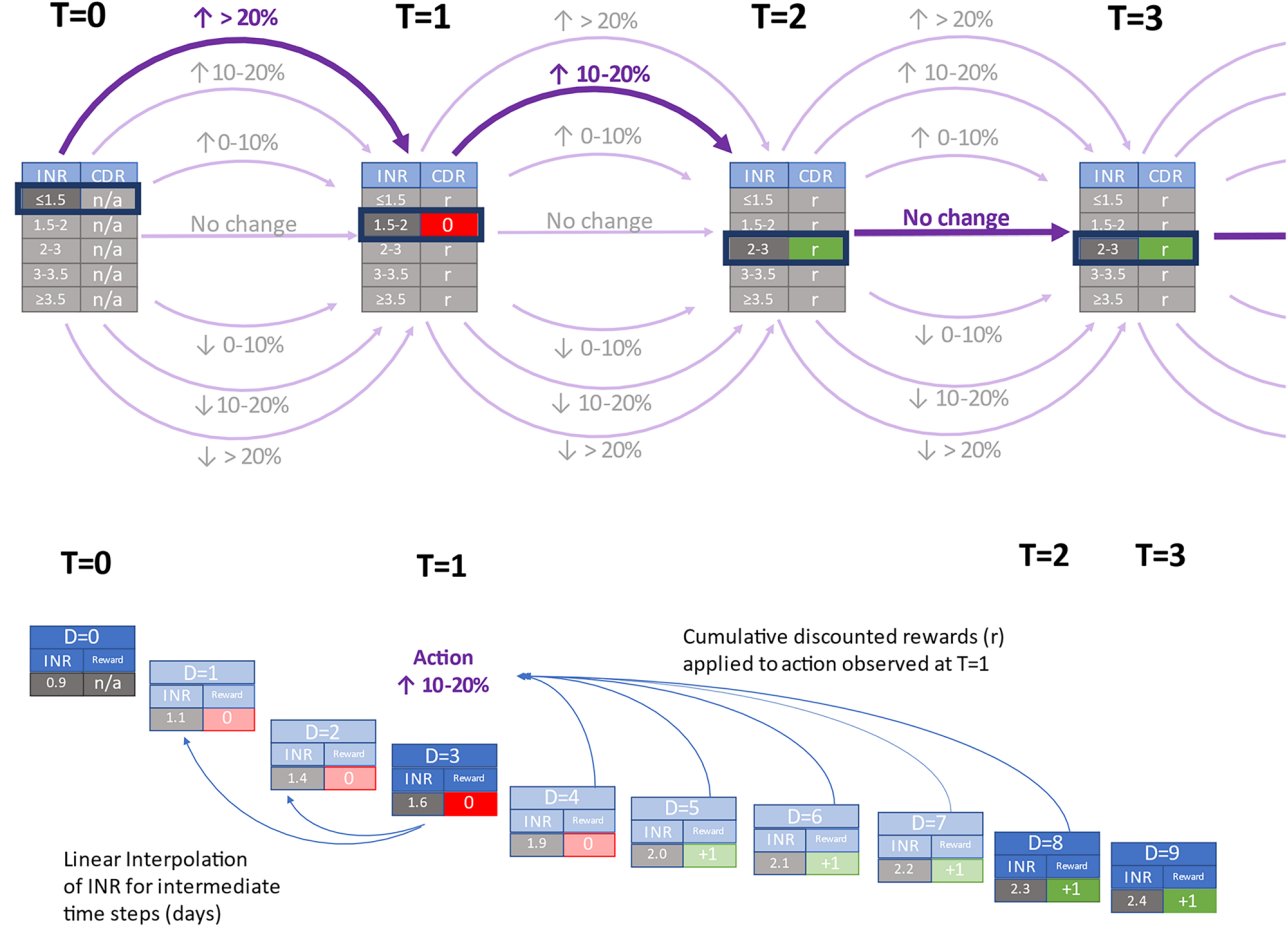
Warfarin dosing optimization as a sequential reinforcement learning task using semi-Markov Decision Processes. The figure illustrates the first several time intervals of a patient trajectory. The highlighted values in the top part of the figure illustrate a single patient trajectory. Each observed time step (T = 0, T = 1, etc.) has a corresponding International Normalized Ratio (INR) test result. The action space is illustrated in purple and is defined as percent changes in warfarin dose—the arrows illustrate that the effect of the change in dose is observed at the following time step. The dynamic elements of the state space are INR test results. We employ a semi-Markov Decision Process framework to handle the inconsistent time intervals between observations inherent to warfarin management, illustrated in the bottom part of the figure. Linear interpolation (Rosendaal’s method) is used to generate INR values for intermediate unobserved time steps. We then calculate intermediate rewards based on interpolated INR values (rewarded when INR is in the range of 2–3) and apply a cumulative discounted rewards to each observed time step. The cumulative discounted reward for an observed time step is applied to the action taken at the previous observed time step (e.g., the dosing action observed at T = 1 is rewarded based on the INR value observed at T = 2). We do not model warfarin initiation, so there is no reward function at T = 0. Observed time step are displayed in darker shades; unobserved interpolated time steps are displayed in lighter shades.

The primary objective of this study was to assess whether a reinforcement learning model for warfarin dosing could achieve equivalent time in therapeutic range and event reduction compared to a previously published clinical algorithm^9^ (referred to throughout as the “benchmark algorithm”). The secondary objective was to assess whether the inclusion of variables associated with warfarin metabolism (such as age, ethnicity, concurrent medications, etc.) improve performance over the traditional approach of focusing more strictly on INR.

## Methods (online supplement)

### Data sources

We developed and validated our model using data from randomized control trials that compared novel oral anticoagulants vs. warfarin. These trials were:

- ARISTOTLE: patients randomized to apixaban (n = 9120) or warfarin (n = 9081) with a median follow up of 21.7 (16.1–28.0) months^30^.
- ENGAGE AF-TIMI 48: patients randomized to higher dose edoxaban regimen (n = 7035), lower dose regimen edoxaban (n = 7034), or warfarin (n = 7036) with a median follow up of 33.6 (29.4–38.4) months^29^.
- ROCKET AF: patients randomized to rivaroxaban (n = 7131) or warfarin (N = 7133) with a median follow up for 22.00 (15.8–27.8) months^31^.
- RE-LY: patient randomized to higher dose dabigatran (n = 6076), lower dose dabigatran (n = 6015), or warfarin (n = 6022) with a median follow up for 24.0 months^28^.

### Patient cohort

Patients in all four trials had atrial fibrillation confirmed by electrocardiogram. All trials excluded patients who had rheumatic mitral stenosis, mechanical prosthetic valves, severe renal impairment, or recent major bleeding, or whose atrial fibrillation was due to a reversible condition. All four trials used an INR target of 2–3 for their warfarin arm. Patients on warfarin were followed for a median of 22.2 months. A difference between patients across the trials was the number of risk factors for stroke required for inclusion^32^.

### Exclusion criteria

We began with 29,272 patients in our combined data set. We excluded all patients who had no recorded dose(warfarin)-response(INR) pairs, as well as all patients with a weekly dose of > 140 mg (well beyond the reasonable range and indicative of data entry errors). For the external validation set, we also excluded any patients who were missing covariates required for performance estimation (described below), leaving a total of 28,232 patients.

### Data preprocessing

For all trials, we extracted patients in the warfarin arms. To ensure each warfarin dose recorded would have a corresponding INR measurement, we removed all warfarin doses recorded before a patient’s first INR measurement and after their last INR measurement.

We extracted a subset of data from the trials based on clinical expert input about the features most relevant to warfarin dosing decisions. We extracted the following features for inclusion in our study: age, sex, weight, region, smoking status, concomitant medications (aspirin/amiodarone/thienopyridines), diabetes status, history of congestive heart failure, history of hypertension, history of myocardial infarction, current absolute warfarin dose, current INR, previous 4 INRs, whether the patient experienced a stroke during the study period, whether the patient experienced a major bleed during the study period, and whether the patient was hospitalized during the study period.

We discretized some continuous variables, which is known to aid in the learning process. These include age, weight, and warfarin dose, with the resulting categorical variable one-hot encoded with the first level dropped (i.e. we created binary dummy variables for each level of the category).

There were differences across the trials in how warfarin doses were recorded, so we normalized to weekly doses since this is reflective of how warfarin is prescribed in clinical practice.

The action space was constructed by categorizing warfarin dose adjustments based on clinical expert input and reference to existing clinical algorithms^8,9^. Possible actions were: decrease > 20%, decrease 10–20%, decrease ≥ 10%, no change, increase ≤ 10%, increase 10–20%, and increase > 20%.

Patient trajectories were organized as longitudinal sequences of dose (warfarin)-response (INR) pairs. We elected to split patient trajectories in a few scenarios. The first is when a significant adverse event was recorded (ischemic stroke, hemorrhagic stroke, major bleeding, hospitalization), since our RL model is meant to inform the management of atrial fibrillation in the community, not acute stroke or hemorrhage. We also split trajectories in cases where more than 90 days passed between clinical visits, since in these cases patients may be receiving care outside of the clinical trial (due to international travel, for example) and thus we deemed imputation unreliable in this context. Thanks to the rigor of data collection for randomized control trials, these data sets required minimal data cleaning, however some missing or impossible values for warfarin dose and INR were identified. In these cases, we elected to split patient trajectories rather than to impute values for the missing data, because imputing values for either feature could result in cases where our model received incorrect rewards, introducing noise into our learned policy. Given the length of patient trajectories and low level of missingness in this data set, we determined that splitting trajectories in these cases would not negatively impact the learning process. Impossible values (such as INR = 0) were treated as missing. After implementing these trajectory splits, we were left with 42,384 patient trajectories for development and evaluation.

### Model development

Our RL model was developed with the Batch Constrained Q-learning algorithm^27^ using a formulation designed for discrete action spaces^33^. The Batch Constrained Q-learning algorithm employs double deep Q-learning^34,35^, accompanied by a generative network to constrain the action space to actions previously observed. We selected this algorithm for its robustness in an “offline” setting where there are suboptimal clinical decisions in the observed data set and where complex pharmacodynamics of warfarin introduce a degree of uncertainty in predicting patient response to dose changes.

Reinforcement learning models, including the Batch Constrained Q-learning algorithm, typically structure the interaction between an agent and its environment as a Markov decision process, which assumes that state transitions occur at regularly spaced timesteps. However, in clinical practice warfarin dosing decisions are made at irregular timesteps. To account for this, we model our decision problem as a semi-Markov decision process^26,36^.

In regular clinical practice, warfarin dose decisions are made during a clinic or virtual visit, and that dose is maintained until the next visit, when a new INR result is available. Using the semi-Markov decision process framework, we model these clinical decisions as “options”, which are sequences of “primitive actions” which we model as daily dose adjustments. For example, if a clinician prescribes a dose adjustment of "Increase by > 20%" on day 5, and the next visit does not occur until day 10, we model this as an option consisting of the primitive action sequence “Increase > 20%” on day 5, and “no change” on days 6–9.

In this framework, the reward for each option is derived from rewards for each primitive action. We use Rosendaal’s linear interpolation method^37^ to generate INR values to reward each primitive action in an option (+ 1 for INR in range, 0 for INR out of range), which are used to calculate a cumulative discounted reward for each option (Fig. 1).

Using this framework, the goal of the Batch Constrained Q-learning algorithm is to the learn the policy

$$\uppi \left(s\right)={\text{argmax}}_{o\in \mathcal{O}\left(s\right)}Q\left(s,o\right)$$

which maps the current patient state $s$ to the best available option $o$ according to its expected return $Q\left(s,o\right)$. The ${\text{argmax}}$ function returns the option $o$ from the set $\mathcal{O}\left({\text{s}}\right)$ with the maximum value of $Q\left(s,o\right)$, where $\mathcal{O}\left({\text{s}}\right)$ is the set of options provided by the generative network for state $s$. The policy $\uppi \left(s\right)$ is learned by optimizing the estimate of the expected return via stochastic gradient descent. The learning process was governed by the following hyperparameters: batch size, learning rate, threshold, number of hidden layers, dimensionality of hidden layers, and the moving average parameter for the value estimator. Hyperparameters were optimized using grid search.

Using this RL framework, we developed a model using current and previous INR values and warfarin doses as model inputs (ie. patient state). For the secondary objective we developed a second model that made use of all of the clinical features described in the previous section.

### Implementation of the benchmark algorithm and non-optimal policies

Comparing a RL policy against observed clinician behavior can lead to overly optimistic assessments of model performance^33,34^. To address this, we implemented three additional policies to evaluate our RL policy against.

The first of these was a clinical algorithm that provides clear warfarin dosing rules based on INR thresholds: if INR is less than 1.5 then increase warfarin dose by 15%, if INR 1.5–1.99 then increase by 10%; if INR 2.0–3.0 then no change, if INR 3.0–4.0 then reduce dose by 10%, if INR 4.0–4.99 then hold the dose for one day and then decrease by 10%, and if the INR was greater than 5.00 the dose was to be held until the INR was therapeutic and then decrease by 15%. Use of this type of algorithm has been shown to provide significant improvement in TTR over un-assisted clinician decision making^9,35^, and thus we refer to it throughout as the “benchmark algorithm.”

We also implemented two intentionally non-optimal policies against which to compare both the benchmark algorithm and our RL policy. One of these is a “no change” policy, which always maintains the current warfarin dose, regardless of changes in INR. The second is a “random” policy, which randomly selects a dose change at each observed time step, with the available options weighted based on their observed frequency in our data set.

### Performance evaluation

To evaluate the relationship between algorithm-consistent dosing and TTR, we fit weighted linear regression models at the center level. Center-level algorithm-consistency was calculated by averaging the difference between the observed warfarin dose and the dose recommended under each policy. Center-level TTR was calculated by averaging the values obtained from patients in each center. Models were weighted by the number of patients per center, and were developed with mean center algorithm-consistency as a predictor and mean center TTR as the outcome. A coefficient of determination (R^2^) was reported for each model.

To estimate the relationship between center-level algorithm-consistency and TTR while controlling for patient, center and country-level factors, we developed multilevel, multivariable linear regression models, with patients nested in centers, and centers nested in countries. Patient-level characteristics included age (years); weight (kg); sex (male vs. female); ethnicity (white vs. other); current smoking status (yes vs. no); history of heart failure (yes vs. no), hypertension (yes vs. no), diabetes mellitus (yes vs. no), stroke (yes vs. no); previous warfarin use (yes vs. no); current amiodarone use (yes vs. no); and current insulin use (yes vs. no). Center-level characteristics included specialty (anticoagulation clinic vs. other), setting (secondary/tertiary hospital vs. primary), and center-level mean algorithm-consistent warfarin dosing (%). Country-level characteristics included the 2006 country Gross Domestic Product (high income vs. medium/low income), Disability Adjusted Life Expectancy (years), and Health System Performance Index. We also included random intercept for center and country.

Finally, to estimate the association between algorithm-consistent dosing and the composite outcome of stroke, systemic embolism, or major hemorrhage we developed multilevel, multivariable Cox proportionate hazard models. The Cox models were adjusted for the same patient-, center-, and country-level characteristics as in the previous multilevel model, including random intercepts. Additionally, four medication variables were included, which were baseline use of aspirin (yes vs. no), β-blockers (yes vs. no), ACE-inhibitors (yes vs. no), or statins (yes vs. no). Because the effect of warfarin dosing on clinical outcomes is mediated through INR control, TTR was not included as a covariate in the Cox models. Patients who experienced stroke, systemic embolism, or major hemorrhage were censored when the first adverse event happened, and algorithm-consistency was calculated using warfarin doses before their first adverse event; for patients who did not experience an event, algorithm consistency was calculated using all warfarin doses throughout the study. Algorithm-consistency was calculated for each center and was analyzed as a center-level variable.

To evaluate whether algorithm consistent dosing at the center-level was a marker of generalized high quality care, independent of its influence on warfarin control, we refit the multilevel Cox proportional hazards model on patients taking dabigatran to assess whether center-level algorithm consistency could independently predict center-level outcomes for dabigatran patients.

### Ethics

This paper is a sub-study of COMBINE AF^32^, approved by the Duke University Institutional Review Board. Additionally, each of the four randomized control trials used in this study had institutional review board approval^28–31^. All participants in these trials provided informed consent, which included consent for secondary use of trial data in future research.

## Results

A total of 28,232 patients with atrial fibrillation from the warfarin arms of four clinical trials of novel oral anticoagulants vs. warfarin were included in our study. The development set was made up 22,502 patients from 52 countries enrolled in the ARISTOTLE, ENGAGE AF-TIMI 48 and ROCKET AF trials. External validation set was made up of 5,730 patients from 44 countries enrolled in the RE-LY trial. All countries included in the external validation set were represented in the development set. The studies were well balanced with respect to most baseline patient characteristics (Table 1). Studies varied slightly in the distribution of continent (RE-LY had more patients from North America and Western Europe, for example) and ethnicity (RELY and ENGAGE have relatively few patients who identified as Hispanic). The most significant difference between the studies was the relatively higher rates of comorbidities in ENGAGE and ROCKET (mean CHADS2 score of 2.8 and 3.5 respectively), vs. ARISTOTLE and RE-LY (CHADS_2_ scores of 2.1), which was due to differences in the number of risk factors for stroke required for inclusion in each trial (1 for RE-LY and ARISTOTLE, 2 or more for ENGAGE and ROCKET).

**Table 1:** Baseline patient characteristics, adverse event rates and study characteristics by trial.

	Development (Train/Tune)			External validation
	ARISTOTLE (n = 8664)	ENGAGE AF-TIMI 48 (n = 6898)	ROCKET AF (n = 6940)	RE-LY (n = 5730)
Baseline characteristics				
Age	69.0 (9.7)	70.5 (9.4)	71.1 (9.4)	71.6 (8.5)
Female (%)	34.9	37.4	39.3	36.1
Region (%)				
East Asia	11	10.3	10.4	12.1
Eastern Europe	23.6	35.9	39.5	15.5
Latin America	19	12.6	13.1	5.2
North America	24.8	22.3	19	36.4
South Asia	3.3	3.3	1.8	2.9
Western Europe	18.3	15.5	16.2	27.8
Race (%)				
Asian	14.6	13.7	12.3	15.5
Black	1.1	1.2	1.1	1.1
Other	1.7	4.0	2.8	8.9
White	82.6	81.1	83.7	74.5
Hispanic (%)	19.9	0.6	16.5	4.7
Weight (kg)	84.3 (20.7)	83.8 (20.1)	81.8 (19.0)	82.9 (19.7)
Systolic BP (mmHg)	131.3 (16.5)	130.0 (15.2)	132.2 (16.2)	131.2 (17.3)
BMI	29.4 (6.1)	29.5 (5.9)	29.0 (5.7)	28.8 (5.8)
Diabetes (%)	25.1	35.9	39.5	23.4
Hypertension (%)	87.8	93.7	90.7	78.7
CAD (%)	32.6	33.5	23.4	27.8
MI (%)	14	11.8	18	16.1
CABG (%)	6.3	6.9	7.2	*N/A*
PCI (%)	9.3	6.8	8.8	*N/A*
HF (%)	35.4	57.5	62.2	31.8
Stroke or TIA (%)	16.9	28.6	51.8	20.0
Paroxysmal AF (%)	15.4	25.3	17.1	33.6
Ever smoked (%)	47.5	40.6	32.8	50.7
CHADS_2_ score	2.1 (1.1)	2.8 (1.0)	3.5 (0.9)	2.1 (1.1)
CHADS_2_ = 1 (%)	33.2	0.1	0	28.5
CHADS_2_ = 2 (%)	36.4	46.9	13.2	36.9
CHADS_2_ > = 3 (%)	29.8	53	86.8	32
Prior VKA use (%)	67	73.7	62.4	68
Aspirin (%)	30.9	29.5	36.4	40.3
Thienopyridines (%)	2	2.3	1.8	6
Beta blockers (%)	63.4	66.2	65.4	62
Calcium channel blockers (%)	30.3	30.6	27.3	32.3
Digoxin (%)	33	29	38.6	28.9
Proton pump inhibitors (%)	13.6	8	12.4	13.7
Creatinine	1.1 (0.3)	1.1 (0.3)	1.1 (0.3)	1.5 (6.6)
Adverse events (% per patient-year)				
Death	2.35	1.57	3.21	1.29
Ischemic stroke	0.82	0.95	1.70	0.85
Major bleeding	3.42	3.54	3.64	3.09
Minor bleeding	9.16	6.85	21.46	30.65
Hemorrhagic stroke	0.49	0.48	0.54	0.37
Hospitalization	20.24	32.61	9.92	37.21
Study characteristics				
Number of trajectories	11,133	11,425	8,254	11,539
Trajectory length in days	459.8 (309.6)	502.8 (400.6)	487.6 (303.3)	293.7 (258.4)
Number of INRs per trajectory	29.5 (13.6)	38.5 (17.2)	26.8 (13.3)	28.9 (14.7)
TTR (%)	55.9	58.1	54.8	61.7

Continuous variables listed as mean (standard deviation). Categorical variables listed as %. Likely erroneous values/outliers (described in “methods” section) were not included in calculations for weight, body mass index, or creatinine clearance. BMI = body mass index, CABG = coronary artery bypass graft, CAD = coronary artery disease, MI = myocardial infarction, NSAID = non-steroidal anti-inflammatory, PCI = percutaneous coronary intervention, PPI = proton pump inhibitor, TIA = transient ischemic attack, VKA = vitamin K antagonist.

**Figure 2:**
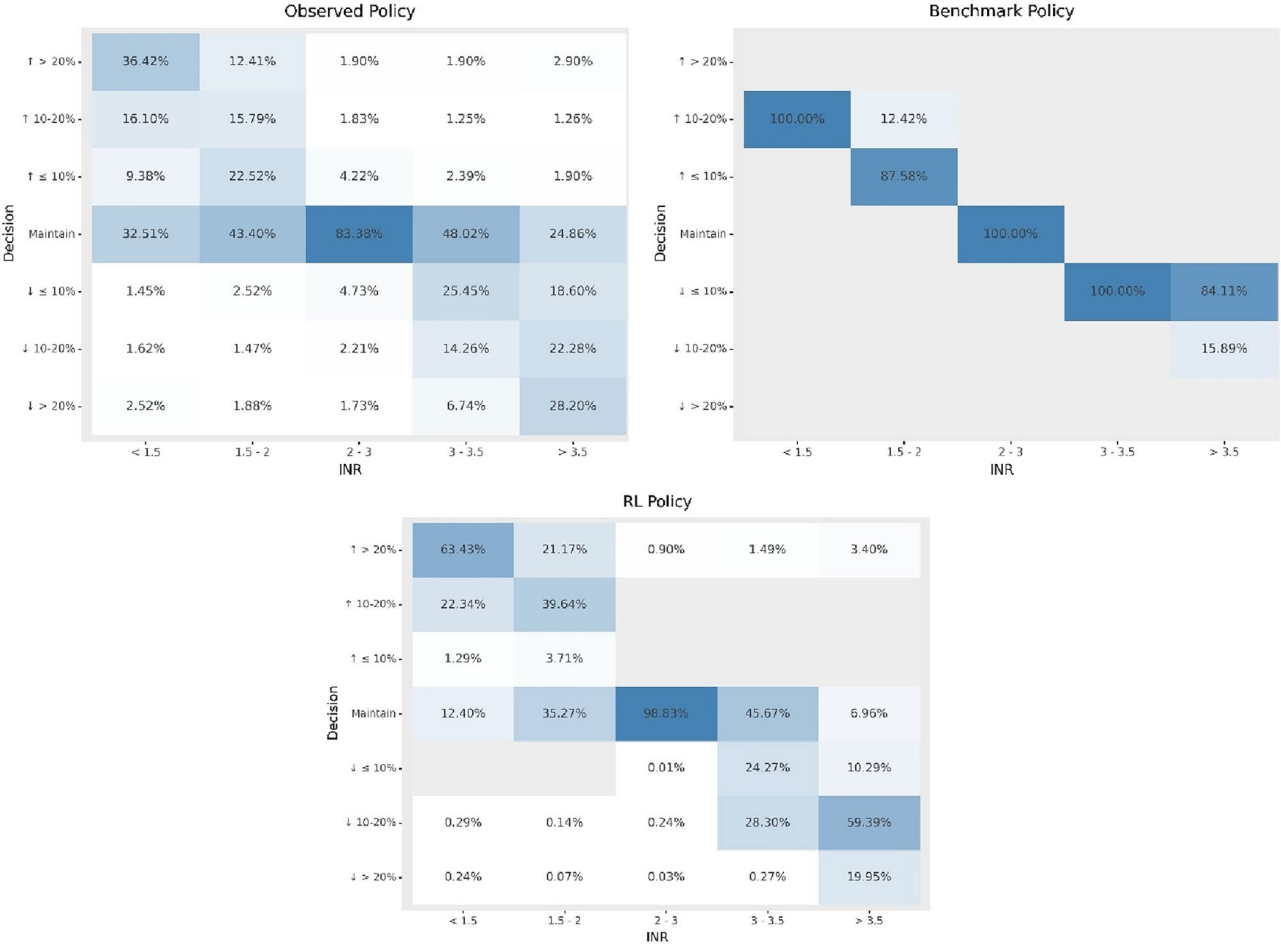
Comparison of clinician, reinforcement learning (RL), and benchmark policies. The figure illustrates clinician, benchmark algorithm and RL policies using heatmaps, where the X axis illustrates INR result bins, and the Y axis illustrates the distribution of dose recommendations within each INR result bin. On average, the RL policy recommends larger dose changes in the context of very high and very low INR values than either the clinician or benchmark policies.

Policies based on observed clinician behavior, the benchmark algorithm and our RL model are visualized in Fig. 2. Observed clinician behavior suggests some clinicians tend to maintain a dose even when INR well out of range (clinicians maintained the dose 32% of the time when INR was < 1.5 and 25% of the time when INR was > 3.5), while others will make relatively large dose adjustments in the same circumstances (36% of the time clinicians increased the dose by > 20% when INR was < 1.5 and 28% of the time decreased the dose by > 20% when the INR was > 3.5). Observed clinical behavior appears to include examples of clinically unintuitive behavior, such as cases where clinicians decrease the dose dramatically when INR was < 1.5 and increased it substantially when INR was > 3.5. Upon reviewing these cases, we found them to be clinical scenarios in which a clinician had temporarily suspended and then resumed a patient’s warfarin dose in response to an extreme INR or a clinical event such as an emergency surgery. The benchmark algorithm provides dosing recommendations based purely on INR thresholds, and so produces a very clean policy with no apparently unintuitive recommendations. The benchmark algorithm was designed to minimize dramatic swings in INR and so does not recommend any dose changes greater than 15%, even when INR is well out of range. The RL policy appears to be more aggressive than the others, rarely maintaining a dose when the INR is out of range and recommending dosage adjustments of > 20% relatively frequently (63% of cases where INR was < 1.5 and 20% of cases where INR was > 3.5). We conducted a manual post hoc review of cases where the RL policy appears to offer unintuitive dose adjustments when INR out of range and found that these were examples of the same temporary warfarin suspension/resumption scenarios described above.

**Figure 3:**
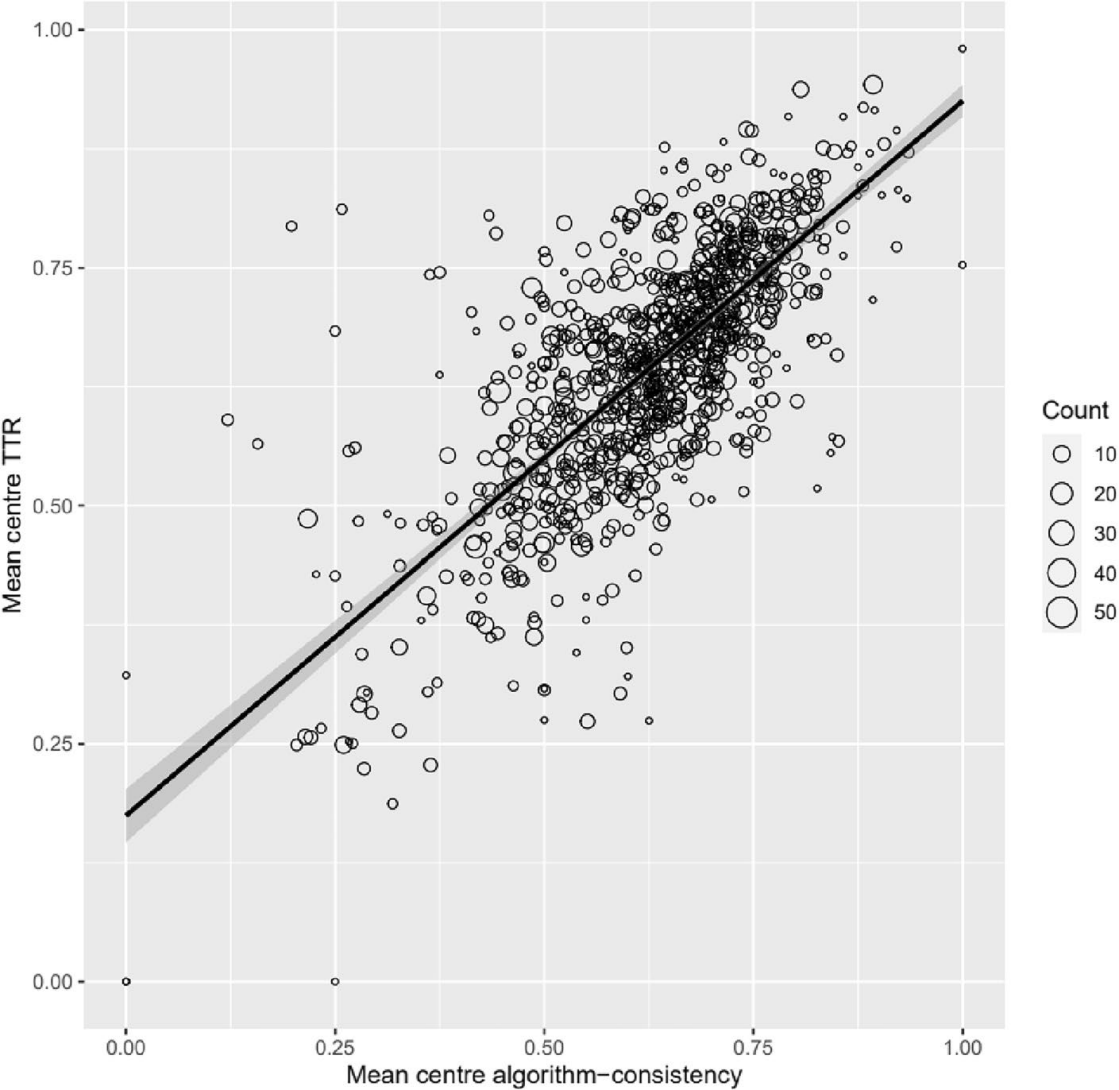
Weighted linear regression of the association between mean center algorithm-consistency and mean center time in therapeutic range (TTR). Mean center algorithm-consistent dosing and TTR were calculated by averaging the values obtained from patients in each center. The regression model was weighted by the number of patients per center. Each data point represents a single center, and the size of the data point represents the number of patients in that center.

We evaluated the potential effectiveness of the RL policy to improve TTR using a weighted linear regression of the association between mean center algorithm agreement and mean center TTR. This analysis was conducted on our external validation set, which included 5730 patients from 903 centers spread across 44 countries. This analysis showed a positive association between RL-consistent dosing and TTR at the center-level (R^2^ = 0.56). We carried out the same analysis for the benchmark algorithm and found a similarly strong association between algorithm consistent dosing and TTR (R^2^ = 0.53) that is consistent with previous research^9^ (Fig. 3).

We further explored the relationship between algorithm consistent dosing and TTR using a multilevel, multivariable linear regression, with patients nested in centers, and centers nested in countries (Table 2). After adjusting for patient, center, and country characteristics, center-level RL-consistent dosing was the strongest predictor of TTR, with each 10% increase in algorithm-consistent dosing at the center level predicting a 6.78% improvement in TTR (95% CI 6.29, 7.28, *p* < 0.001). We repeated this analysis for the benchmark algorithm and found comparable results, with each 10% increase in algorithm-consistent dosing predicting a 6.10% increase in TTR (95% CI 5.67, 6.54, *p* < 0.001).

**Table 2:** Multilevel multivariable linear regression model for patient-level time in therapeutic range.

Characteristics	Adjusted change in mean TTR	95% CI	*p* value
Patient-level			
Age (per year)	− 0.02	− 0.08, 0.04	0.594
Weight (per kg)	0.02	− 0.01, 0.05	0.187
Male	1.11	0.08, 2.14	0.035
White	2.32	0.84, 3.85	0.003
Current smoker	− 4.24	− 6.03, − 2.43	< *0.001*
History of heart failure	− 2.33	− 3.39, − 1.26	< *0.001*
History of hypertension	− 0.02	− 1.18, 1.16	0.979
History of diabetes mellitus	− 1.26	− 2.45, − 0.08	0.038
Previous stroke	0.01	− 1.43, 1.45	0.993
Previous warfarin use	2.54	1.57, 3.48	< *0.001*
Current amiodarone use	− 1.85	− 3.35, − 0.34	0.016
Current insulin use	− 3.03	− 5.40, − 0.67	0.012
Center-level			
RL-consistent dosing (per 10%)	*6.78*	*6.29, 7.28*	< *0.001*
Secondary/tertiary hospital	1.28	− 0.22, 2.75	0.093
Anticoagulation clinic	0.29	− 1.17, 1.74	0.698
Country-level			
High income	2.58	− 1.03, 6.18	0.175
Disability Adjusted Life Expectancy	0.14	− 0.36, 0.64	0.605
Health System Performance Index	0.95	− 22.66, 24.64	0.94

To examine the degree to which the improvement in TTR associated with RL-consistent dosing might translate into reductions in stroke and other events, we developed a multilevel, multivariable Cox proportional hazard model to examine the relationship between center-level algorithm-consistency and a composite outcome of stroke, systemic embolism, or major hemorrhage (Table 3). We found that each 10% increase in RL-consistent dosing was associated with a 11% decrease in the composite outcome (HR 0.89; 95% CI 0.81, 0.98, *p* = 0.015). We repeated this analysis for the benchmark algorithm and found that every 10% increase in agreement was associated with an 10% decrease in the composite outcome (HR 0.90; 95% CI 0.83, 0.98, *p* = 0.018).

**Table 3:** Multi-level multivariable cox proportional hazard model for stroke, systemic embolism, or major hemorrhage.

Characteristics	Adjusted hazard ratio	95% CI	*p* value
Patient-level			
Age (per year)	1.03	1.02, 1.05	< 0.001
Weight (per kg)	0.99	0.99, 1.00	0.012
Male	1.1	0.90, 1.36	0.35
White	0.71	0.56, 0.89	0.004
Current smoker	1.31	0.92, 1.87	0.13
History of heart failure	1.23	1.00, 1.52	0.046
History of hypertension	1.3	1.01, 1.67	0.04
History of diabetes mellitus	1.19	0.94, 1.51	0.14
Previous stroke	1.3	1.00, 1.70	0.053
Previous warfarin use	1.04	0.85, 1.27	0.71
Current amiodarone use	0.96	0.69, 1.34	0.82
Current insulin use	1.64	1.11, 2.42	0.013
Baseline use of β-blocker	0.94	0.77, 1.14	0.52
Baseline use of aspirin	1.24	1.01, 1.52	0.036
Baseline use of ace-inhibitor	0.97	0.80, 1.17	0.73
Baseline use of statin	1.09	0.90, 1.33	0.36
Center-level			
RL-consistent dosing (per 10%)	*0.89*	*0.81, 0.98*	*0.015*
Secondary/tertiary hospital	0.82	0.61, 1.09	0.16
Anticoagulation clinic	0.95	0.72, 1.24	0.68
Country-level			
High income	1.72	1.19, 2.50	0.004
Disability Adjusted Life Expectancy	1	0.95, 1.06	0.95
Health System Performance Index	0.11	0.01, 1.37	0.087

The association between algorithmically consistent dosing and the composite clinical outcome suggests a potential causal relationship, but a lower event rate at centers with better anticoagulation management could alternately be the result of generally higher quality care at those centers. However, if the lower composite clinical event rate were the result of higher quality of care at the center-level, then we would expect to see a similarly lower rate of events among dabigatran patients at the same centers. To assess this, we refit the Cox model for dabigatran patients in the evaluation set (N = 9467), but found no association between center-level algorithm consistent dosing and the composite clinical event rate for patients on dabigatran (HR 0.96; 95% CI 0.90, 1.02, *p* = 0.16).

To assess whether the inclusion of variables associated with warfarin metabolism provided any improvement in performance over a more strict focus on INR and previous dose, we used the same multilevel, multivariable regression models to evaluate a second RL model that included clinical features such as age, ethnicity, and concurrent medications in addition to INR and previous warfarin doses. There was very little difference in the performance of these two models, with each 10% increase in algorithm-consistent dosing predicting a 6.78% improvement in TTR in the case of the INR/dose only model and 6.49% in the case of the model that included the additional clinical features. The difference was slightly larger in the case of events, with each 10% increase in algorithm-consistent dosing associated with a 10% decrease in the composite outcome for the INR/dose only model and 7% for the model that included the additional clinical features.

## Discussion

In this study of patients with atrial fibrillation receiving vitamin K antagonist therapy, we demonstrate that a RL model could achieve TTR improvement and event reduction equivalent to an established rules-based warfarin dosing algorithm designed by clinical experts. This data driven model was developed using 3 international randomized control trials, and externally validated on a fourth trial of patients living in 44 countries across 6 continents. In addition to potentially improving warfarin dosing for atrial fibrillation (still being used commonly around the world despite the rise of novel oral anticoagulants) the methodology described in this paper may have important applications in other clinical domains, including dosing of warfarin for other indications (such as patients with mechanical valves) as well as optimization of other medications where dosing is based on sequential dose–response relationships, such as heparin, insulin, oral antihyperglycemic drugs, and phenytoin.

This study helps address some of the existing knowledge gaps with respect to the use of machine learning to improve warfarin dosing. A number of machine learning-based systems have been developed, but many of these have been focused on patients within a single jurisdiction or ethnic group, which may limit their generalizability^41–46^. A number of machine learning studies have leveraged International the Warfarin Pharmacogenetics Consortium dataset of 5700 patients from 21 countries, however many of these have incorporated genetic markers associated with warfarin metabolism^46–50^, which may offer valuable performance improvements, but cannot be easily integrated into routine clinical practice (while single nucleotide polymorphisms CYP2C9 and VKORC1 were available for a subset of our patients, we elected not to use them in order to enhance the generalizability of our model for real world clinical settings, especially low and middle income countries where warfarin is still routinely used). Ours is also the first study we are aware of to evaluate the use of machine learning for the long-term dynamic management of warfarin in the community, versus warfarin initiation or short-term management during a hospital stay^42–47^. Our study is the largest its kind by a considerable margin, which is relevant given the data-hungry nature of machine learning methods.

Our study also reveals that clinical features related to warfarin metabolism do not appear to provide sufficient information to justify their inclusion in a model. While one of the strengths of a machine learning approach is to be able to incorporate high dimensional data, in this case the established clinical approach of including only INR and previous warfarin doses to drive decision making produced the best performing model. While are unable to say definitively what was responsible for this phenomenon, given the nature of neural networks it is likely that it is due to a form of overfitting. In particular, the personalized model may have been learning spurious correlations between demographic features and INR that do not generalize between our development data set and the external validation data set (Table S1).

A common limitation of most reinforcement learning research in the health domain has been the lack of rigorous statistical evaluation. Unlike supervised learning approaches that can be evaluated using standard techniques for classification and regression, evaluation of RL models on observational data have typically been limited to importance sampling or ad hoc methods (such as U-curves), both of which have limitations that can produce performance estimates that are overly-optimistic^19,38,39^. We were able to address this limitation by taking advantage of the multicenter structure of our data to apply statistically robust techniques developed in a previous study of warfarin dosing algorithms^9,40^. This approach allowed us to provide a rigorous estimate the relationship between algorithmically consistent dosing, TTR, and patient outcomes.

Our study has a number of important advantages over some other reinforcement learning applications in health care, owing to the structure of the warfarin dosing problem. In many clinical domains where RL has been applied, such as sepsis, data sets have not always contained all the data relevant to clinical decision making, which can lead to estimates confounded by spurious correlation^38^. In addition, RL studies in domains such as sepsis can suffer from a sparsity of rewards, where events of interest can be so infrequent that it becomes challenging to assess the value of an individual treatment decision^39^. In our case, however, the data set contained all the relevant data points used to manage warfarin in routine clinical practice. Moreover, the causal connection between INR and clinically relevant outcomes is well-established, which allowed us to use INR results to reward (or not) every decision the model observed during training.

Finally, it is important to note that while our study establishes that RL can be used to learn an optimal policy for warfarin dosing, it provides a relatively small improvement over that of a rules-based clinical algorithm. While our study suggests promise for the future of artificial intelligence to learn directly from clinical data to enhance care, it is important to recognize just how effective a well-researched, interpretable rule-based algorithm developed by clinical experts can be in supporting better clinical practice.

### Limitations

Our study is retrospective, so prospective evaluation through a randomized control trial should be undertaken prior to adoption of our model into routine clinical practice. Because our study is based on data from the setting of clinical trials with strict inclusion/exclusion criteria and selected centers, our results may underestimate real-world variation in TTR between centers^51,52^ as well as the association between warfarin dosing and clinical outcomes. While our evaluation methods account for some social factors such as country-level GDP, we were not able to consider factors such as income or education level, which can include warfarin dose variability, TTR and outcomes. Our methodology employs deep neural networks, whose inner workings are black boxes – while explainable machine learning techniques may go some way to increasing the clinical acceptability of black box models, these techniques have some limitations of their own^53^.

### Summary

In summary, we trained a deep reinforcement learning model on longitudinal data from 22,502 patients on warfarin, and externally validated this model on a further 5730 warfarin patients from 44 countries across 6 continents. Our findings suggest that a machine learning algorithm is capable of optimizing time in therapeutic range for patients taking warfarin. A digital clinical decision support system to promote algorithm-consistent warfarin dosing could optimize time in therapeutic range and improve clinical outcomes in atrial fibrillation globally.

## Data availability

Access to the RCT data used for this study is governed by the COMBINE AF steering committee. Investigators interested in working with this data set should contact a member of the steering committee to discuss potential collaborations^32^. The complete source code for this study is publicly available on GitHub^54^. We have deployed the final model for demonstration and validation purposes as a publicly available web tool^55^.

### Supplementary Information

Supplementary Information.
