# Supplementary material for: Optimizing warfarin dosing for patients with atrial fibrillation using machine learning
Source: Sci Rep. 2024 Feb 24;14:4516. doi: 10.1038/s41598-024-55110-9 (PMC10894214; doi:10.1038/s41598-024-55110-9)
Supplement: Supplementary file 1 — Supplementary Information. [file 41598_2024_55110_MOESM1_ESM.docx]

## Supplemental Materials

### Model Development Implementation

Models were developed in Python 3^1^ using PyTorch^2^. Hyperparameter optimization for both models was performed using grid search, implemented in Ray Tune^3^. Model selection used a tuning set split off from the development set consisting of 20% of the patients in ARISTOTLE, randomly selected. The remainder of the development set was used as the training set.

Behavioural cloning models were developed in order to initialize our BCQ-SMDP model and evaluate it using weighted importance-sampling. Candidate behavior cloning models were trained on the training set using the following grid of hyperparameters:

- Number of epochs: 100-500
- Learning rate: 1e-4, 1e-3
- Batch size: 16
- Number of hidden layers: 2
- Dimensionality of hidden layers: 64

over three random seeds. An optimal behavior cloning model for initializing the generative network of the BCQ-SMDP model was selected by evaluating performance in the tuning set according to the multi-class F1 score, which measured how well the network correctly predicted a set of actions which included the observed action. An optimal behavior cloning model for weighted importance-sampling based evaluation of the BCQ-SMDP model was selected by evaluating performance in the tuning set according to the calibration score, which measured how well-calibrated the output probabilities of the behavior cloning network were.

The BCQ-SMDP model was trained on the training set using the following grid of hyperparameters:

- Number of epochs: 50-2500
- Generative network threshold: 0.3, 0.4
- Batch size: 32
- Number of hidden layers: 2
- Dimensionality of hidden layers: 64
- Learning rate: 1e-5
- Moving average update parameter: 5e-3

over two random seeds. The optimal BCQ-SMDP model was selected by the weighted importance sampling estimate of policy value^4^ on the patients from the tuning set.

### Model Evaluation Implementation

The multi-level models for assessing the relationship between algorithm-consistent dosing, TTR, and adverse event rate were implemented in R^5^ using lme4^6^ and coxme^7^.

### Full Comparisons With Baseline and Alternative Models

To fully interrogate the performance of the BCQ-SMDP approach (which uses previous warfarin doses and INR as inputs), we we estimated the multi-level models of time in therapeutic range (TTR) and adverse events for all of the following dosing algorithms: a Random Action model (known to be non-optimal), an Always Maintain model (known to be non-optimal), a Benchmark clinical algorithm (known to improve TTR and outcomes), and two other BCQ-SMDP approaches with different patient features 1) a Personalized model, which incorporated a number of baseline clinical covariates thought to be important to warfarin management to the inputs, 2) an INR only model, which excluded prior warfarin doses from the inputs. These results are summarized in Figures S1 and S2 and Tables S1 and S2.

| A. Random  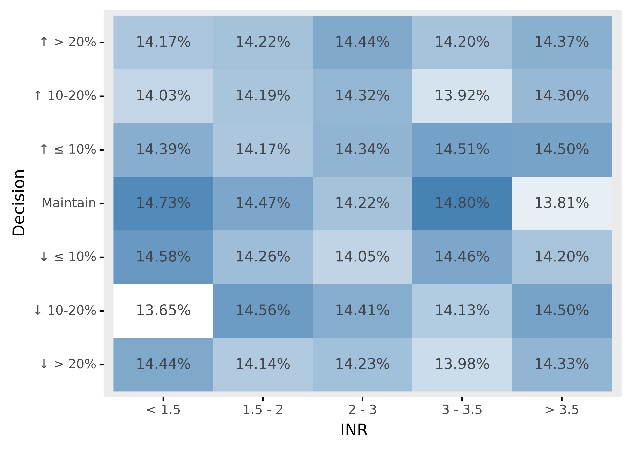 | **B.** Maintain  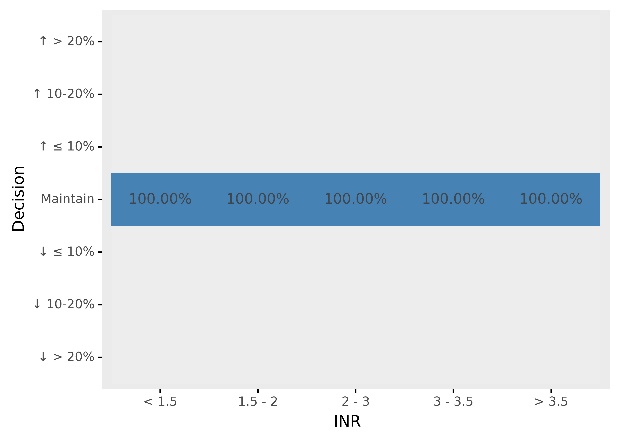 | **C.** Benchmark  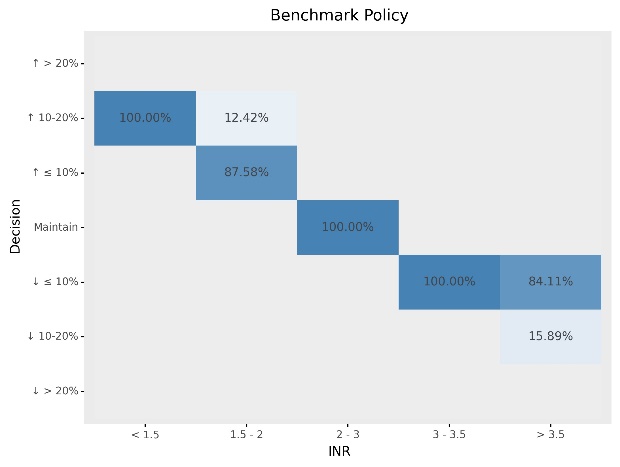 |
| --- | --- | --- |
| D. **BCQ-SMDP (Dose + INR)**  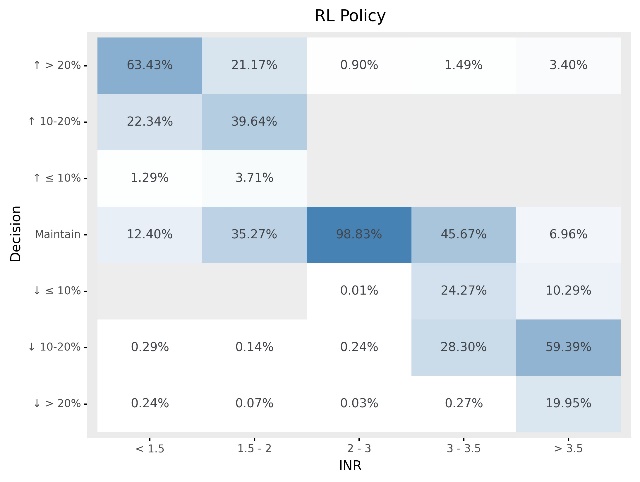 | **E.** BCQ-SMDP (Personalized)  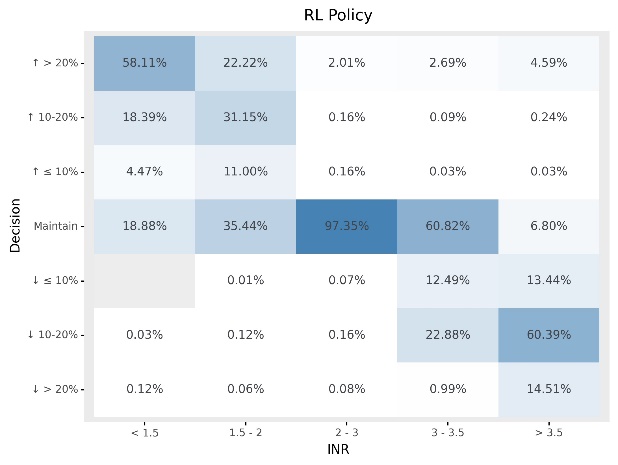 | **F.** BCQ-SMDP (INR Only)  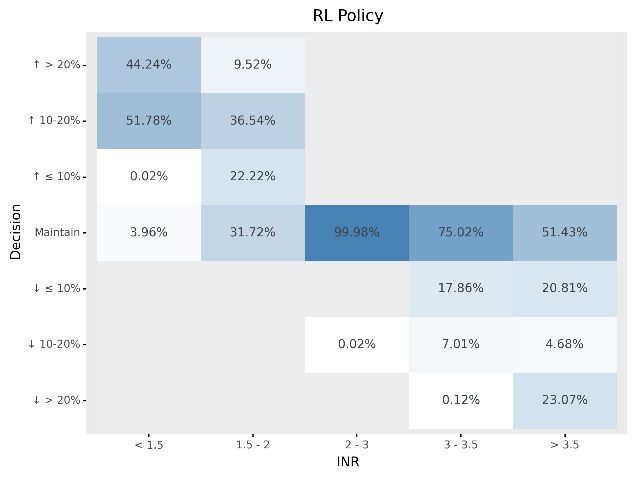 |

**Figure S1. Comparison of all policies’ dosing decisions based on patients’ most recent INR result** *The figure using heatmaps, where the X axis illustrates INR result bins, and the Y axis illustrates the distribution of dose recommendations within each INR result bin.*

| **A.** Random  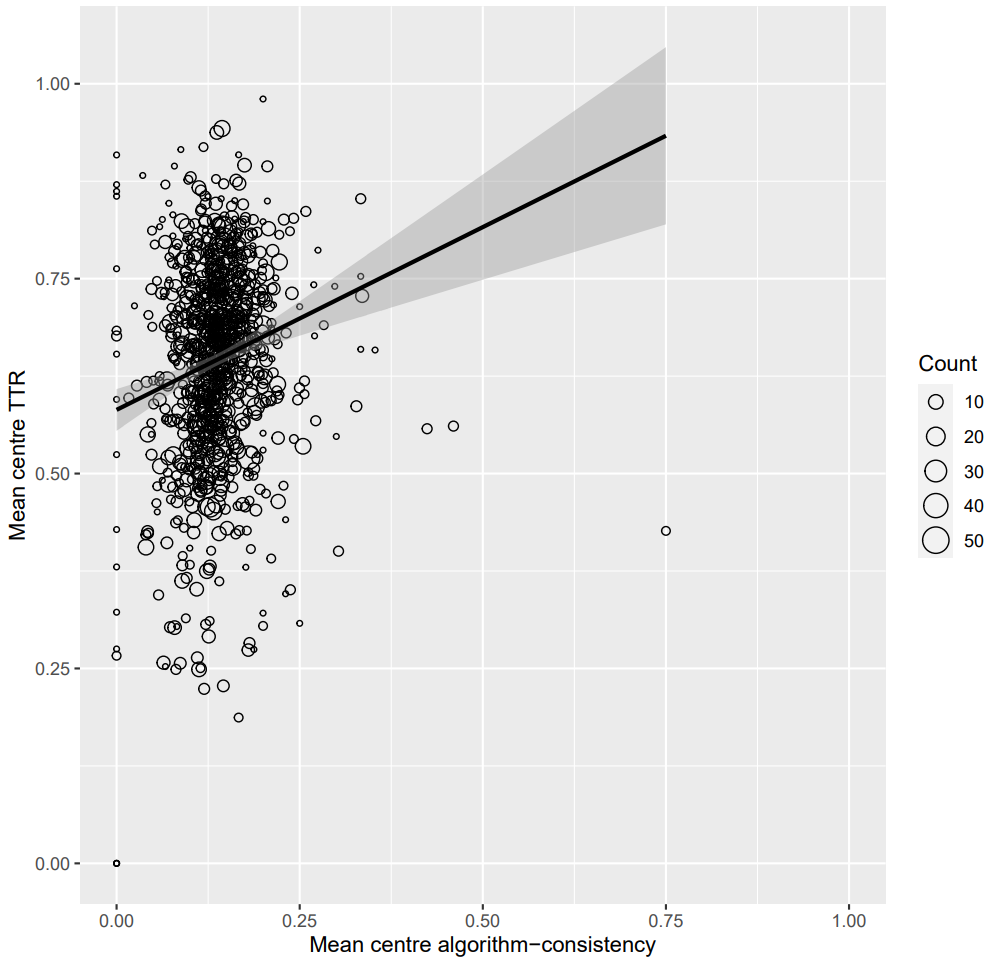 | **B.** Maintain  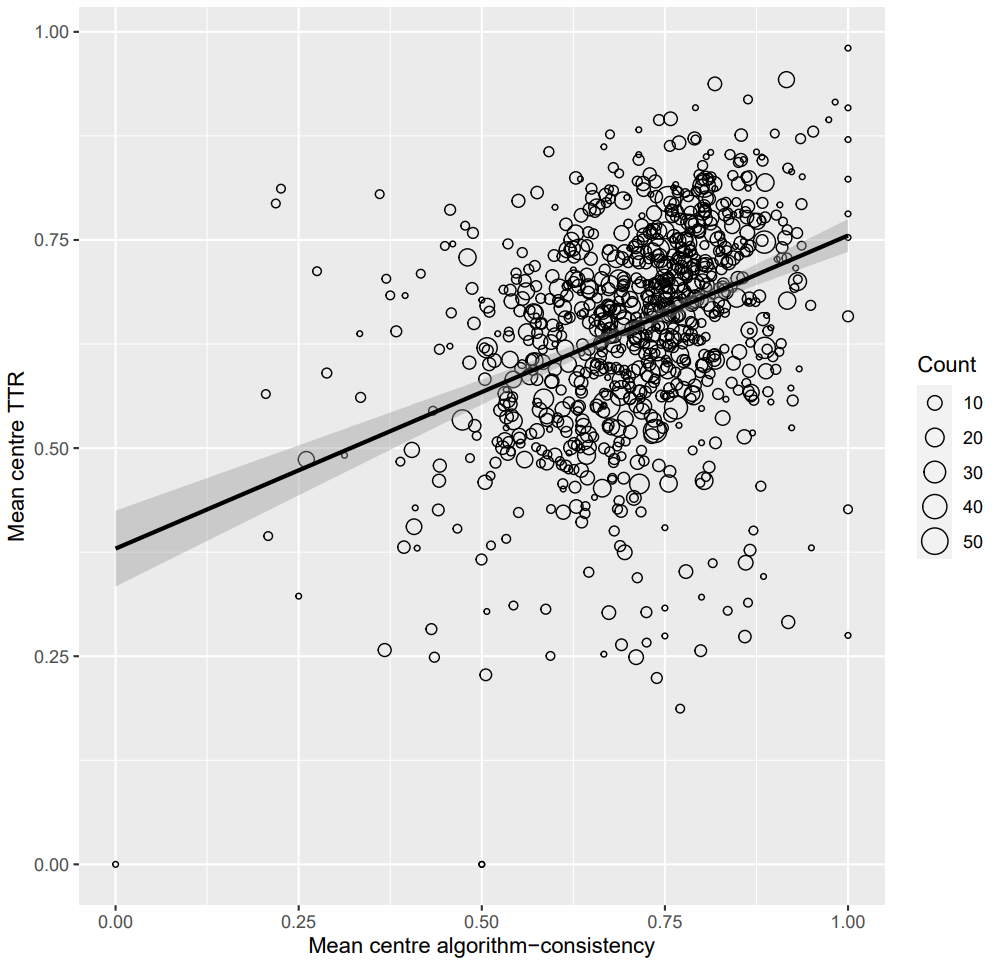 | **C.** Benchmark  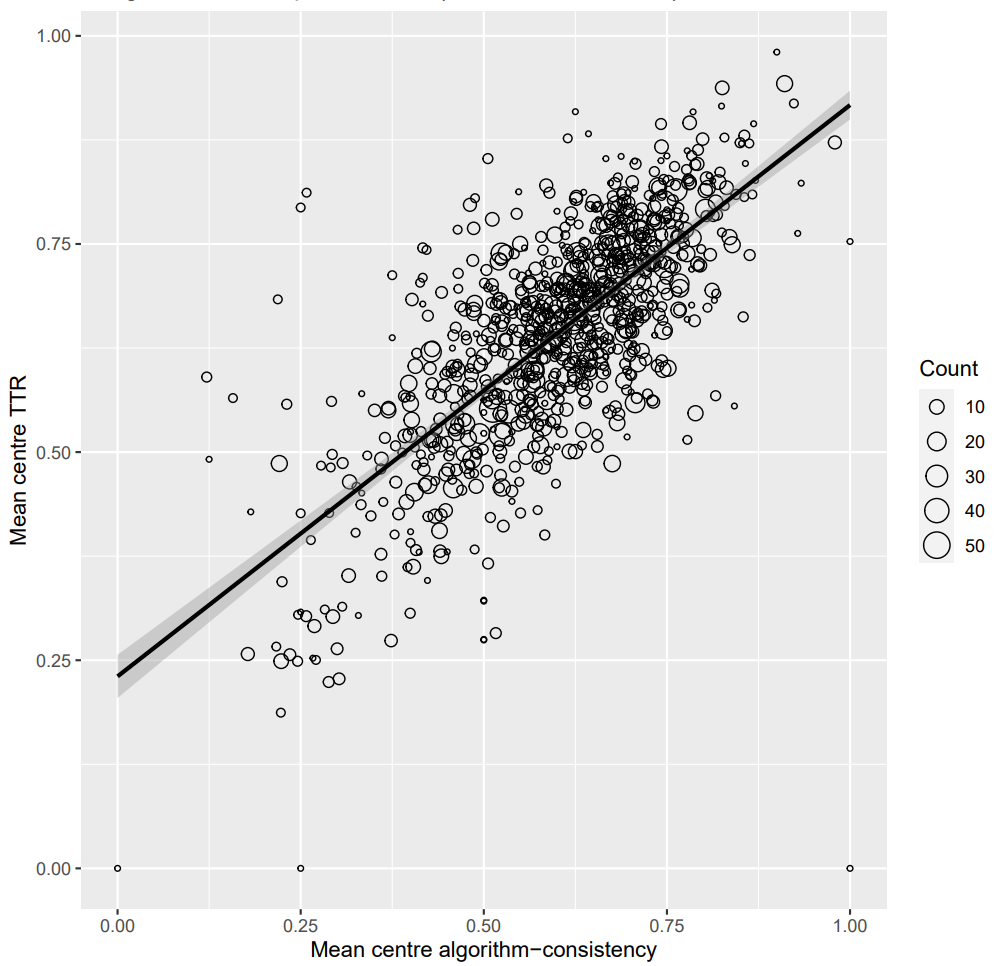 |
| --- | --- | --- |
| **D. BCQ-SMDP (Dose + INR)**  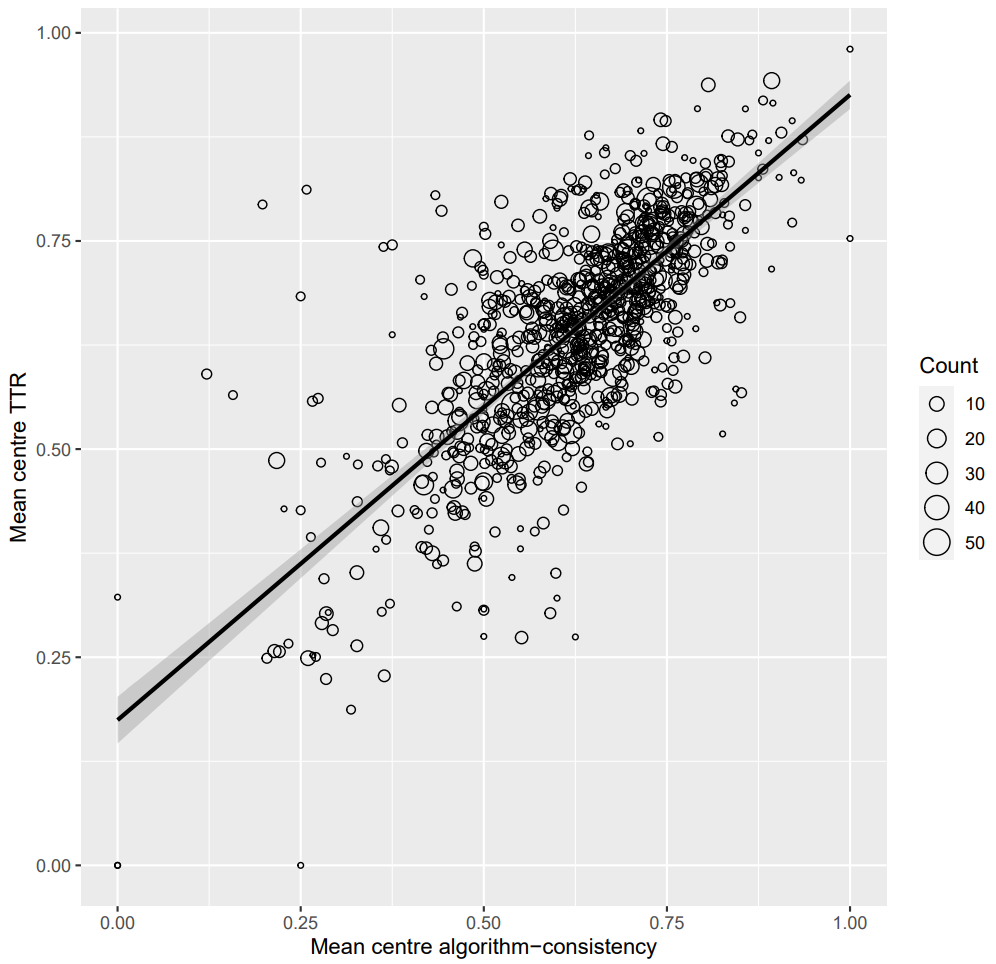 | **E.** BCQ-SMDP (Personalized)  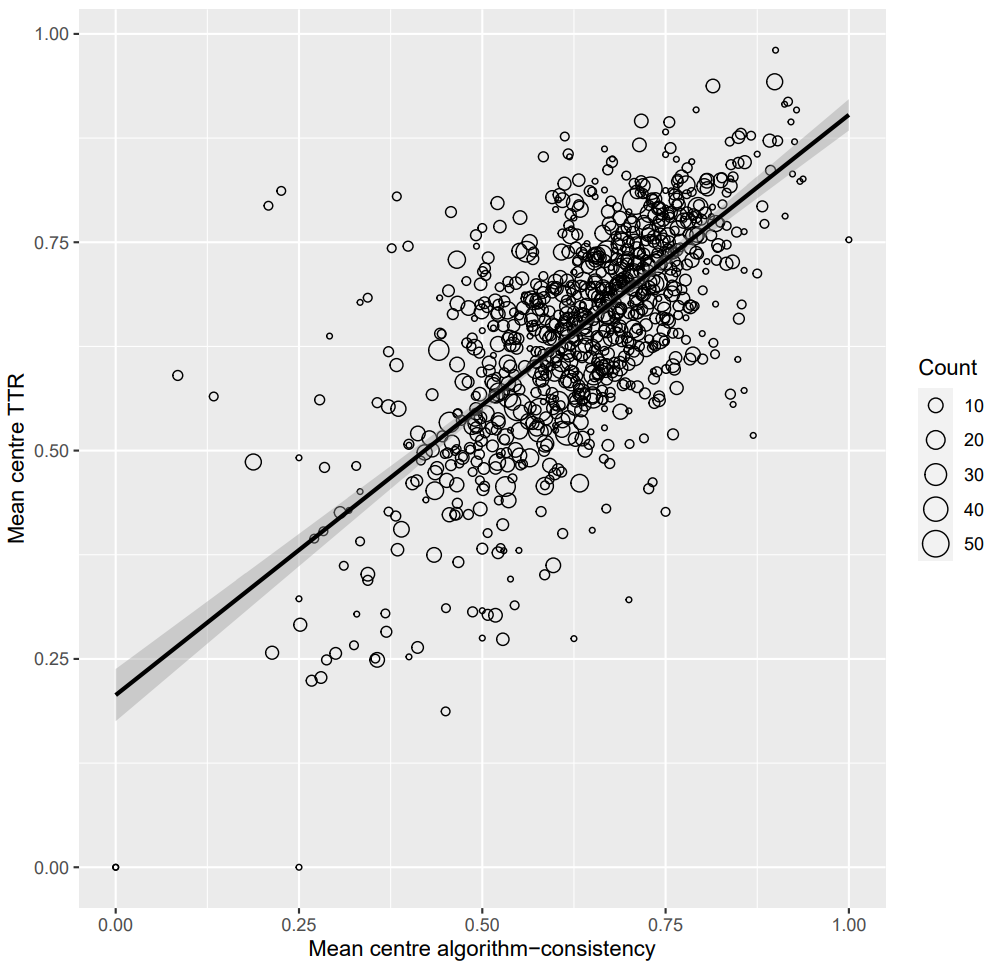 | **F.** BCQ-SMDP (INR Only)  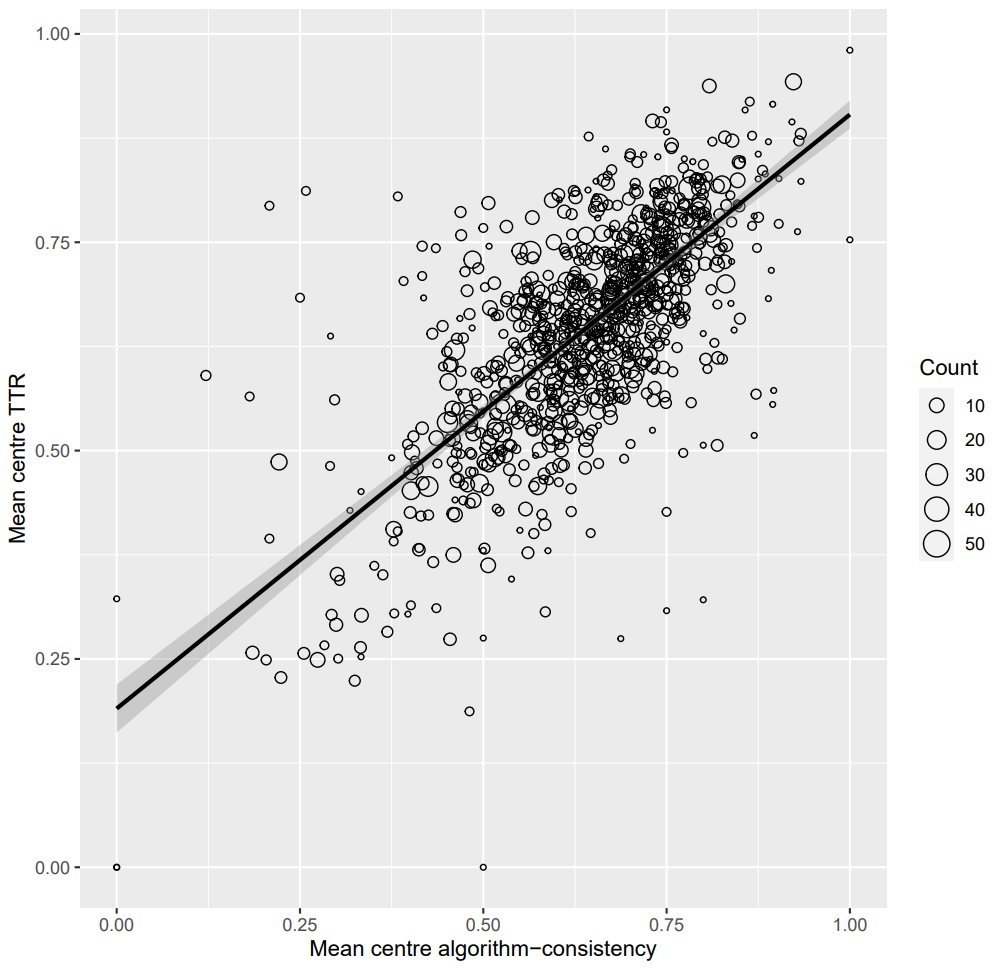 |

***Figure S2. Weighted linear regression of the association between mean center algorithm-consistency and mean center time in therapeutic range (TTR).*** *Each data point represents a single center, and the size of the data point represents the number of patients in that center.*.

**Table S1. Multilevel Multivariable Linear Regression Model for Patient-Level Time in Therapeutic Range**

|  | ***Dosing Algorithm*** | | | | | |
| --- | --- | --- | --- | --- | --- | --- |
| ***Characteristics*** | Random | Maintain | Benchmark | **BCQ-SMDP**  **(Dose + INR)** | BCQ-SMDP  (Personalized) | BCQ-SMDP  (INR Only) |
| **Patient-level** |  |  |  |  |  |  |
| Age (per year) | -0.02 (-0.09, 0.04) | -0.02 (-0.08, 0.04) | -0.03 (-0.09, 0.03) | -0.02 (-0.08, 0.04) | -0.02 (-0.08, 0.05) | -0.02 (-0.09, 0.04) |
| Weight (per kg) | 0.03 (0.00, 0.06) | 0.03 (-0.01, 0.06) | 0.02 (0.00, 0.05) | 0.02 (-0.01, 0.05) | 0.02 (-0.01, 0.05) | 0.02 (-0.01, 0.05) |
| Male | 1.44 (0.37, 2.51) | 1.32 (0.26, 2.39) | 1.06 (0.02, 2.08) | 1.11 (0.08, 2.14) | 1.11 (0.08, 2.14) | 1.09 (0.05, 2.12) |
| White | 3.22 (1.47, 5.00) | 3.37 (1.65, 5.11) | 2.33 (0.90, 3.88) | 2.32 (0.84, 3.85) | 2.45 (0.93, 4.04) | 2.25 (0.72, 3.81) |
| Current smoker | -4.69 (-6.56, -2.82) | -4.68 (-6.54, -2.82) | -4.13 (-5.93, -2.33) | -4.24 (-6.03, -2.43) | -4.57 (-6.37, -2.75) | -4.29 (-6.10, -2.48) |
| History of heart failure | -2.64 (-3.76, -1.51) | -2.64 (-3.75, -1.52) | -2.34 (-3.39, -1.27) | -2.33 (-3.39, -1.26) | -2.37 (-3.43, -1.30) | -2.46 (-3.53, -1.38) |
| History of hypertension | 0.21 (-1.01, 1.43) | 0.21 (-0.99, 1.43) | 0.11 (-1.04, 1.28) | -0.02 (-1.18, 1.16) | 0.19 (-0.98, 1.36) | 0.17 (-0.99, 1.35) |
| History of diabetes mellitus | -1.54 (-2.78, -0.32) | -1.45 (-2.68, -0.23) | -1.37 (-2.56, -0.18) | -1.26 (-2.45, -0.08) | -1.26 (-2.46, -0.08) | -1.22 (-2.42, -0.03) |
| Previous stroke | -0.09 (-1.60, 1.41) | -0.07 (-1.57, 1.43) | -0.07 (-1.51, 1.37) | 0.01 (-1.43, 1.45) | -0.22 (-1.66, 1.23) | -0.12 (-1.57, 1.34) |
| Previous warfarin use | 2.93 (1.93, 3.92) | 2.8 (1.80, 3.78) | 2.75 (1.78, 3.69) | 2.54 (1.57, 3.48) | 2.66 (1.69, 3.61) | 2.49 (1.51, 3.44) |
| Current amiodarone use | -2.29 (-3.88, -0.72) | -2.2 (-3.78, -0.64) | -1.81 (-3.30, -0.31) | -1.85 (-3.35, -0.34) | -1.88 (-3.39, -0.37) | -1.89 (-3.41, -0.38) |
| Current insulin use | -3.42 (-5.86, -0.97) | -3.32 (-5.75, -0.88) | -3.26 (-5.62, -0.90) | -3.03 (-5.40, -0.67) | -2.96 (-5.33, -0.59) | -3.08 (-5.46, -0.70) |
| **Centre-level** |  |  |  |  |  |  |
| *Algorithm-consistent dosing (per 10%)* | 2.41 (0.83, 3.96) | 3.10 (2.47, 3.73) | 6.10 (5.67, 6.54) | 6.78 (6.29, 7.28) | 6.50 (5.99, 7.00) | 6.37 (5.86, 6.88) |
| Secondary/tertiary hospital | 1.73 (-0.36, 3.73) | 2.21 (0.21, 4.12) | 0.43 (-1.06, 1.86) | 1.28 (-0.22, 2.75) | 1.76 (0.19, 3.27) | 1.55 (-0.02, 3.07) |
| Anticoagulation clinic | 1.38 (-0.63, 3.41) | 0.58 (-1.34, 2.53) | 0.68 (-0.77, 2.10) | 0.29 (-1.17, 1.74) | 0.16 (-1.34, 1.67) | -0.11 (-1.63, 1.40) |
| **Country-level** |  |  |  |  |  |  |
| High income | 4.17 (-0.62, 8.94) | 3.60 (-1.10, 8.29) | 3.17 (-0.05, 6.36) | 2.58 (-1.03, 6.18) | 3.31 (-0.69, 7.31) | 2.24 (-1.55, 6.01) |
| Disability Adjusted Life Expectancy | 0.79 (0.13, 1.45) | 0.61 (-0.04, 1.26) | 0.33 (-0.11, 0.78) | 0.14 (-0.36, 0.64) | 0.26 (-0.29, 0.82) | 0.26 (-0.27, 0.78) |
| Health System Performance Index | -27.21 (-58.37, 4.05) | -21.06 (-51.64, 9.60) | -5.6 (-26.61, 15.59) | 0.95 (-22.66, 24.64) | -4.75 (-30.86, 21.44) | -6.59 (-31.27, 18.19) |

**Table S2. Multi-Level Multivariable Cox Proportional Hazard Model for Stroke, Systemic Embolism, or Major Hemorrhage**

|  | ***Dosing Algorithm*** | | | | | |
| --- | --- | --- | --- | --- | --- | --- |
| ***Characteristics*** | Random | Maintain | Benchmark | **BCQ-SMDP**  **(Dose + INR)** | BCQ-SMDP  (Personalized) | BCQ-SMDP  (INR Only) |
| **Patient-level** |  |  |  |  |  |  |
| Age (per year) | 1.03 (1.02, 1.05) | 1.03 (1.02, 1.05) | 1.03 (1.02, 1.05) | 1.03 (1.02, 1.05) | 1.03 (1.02, 1.05) | 1.03 (1.02, 1.05) |
| Weight (per kg) | 0.99 (0.99, 1.00) | 0.99 (0.99, 1.00) | 0.99 (0.99, 1.00) | 0.99 (0.99, 1.00) | 0.99 (0.99, 1.00) | 0.99 (0.99, 1.00) |
| Male | 1.1 (0.89, 1.35) | 1.11 (0.90, 1.36) | 1.11 (0.90, 1.36) | 1.1 (0.90, 1.36) | 1.1 (0.89, 1.36) | 1.11 (0.90, 1.36) |
| White | 0.70 (0.55, 0.89) | 0.70 (0.55, 0.89) | 0.71 (0.56, 0.89) | 0.71 (0.56, 0.89) | 0.7 (0.55, 0.89) | 0.71 (0.56, 0.90) |
| Current smoker | 1.32 (0.93, 1.89) | 1.33 (0.93, 1.89) | 1.31 (0.92, 1.87) | 1.31 (0.92, 1.87) | 1.32 (0.93, 1.88) | 1.32 (0.93, 1.88) |
| History of heart failure | 1.25 (1.01, 1.53) | 1.25 (1.01, 1.53) | 1.23 (1.00, 1.52) | 1.23 (1.00, 1.52) | 1.24 (1.01, 1.52) | 1.23 (1.00, 1.52) |
| History of hypertension | 1.3 (1.01, 1.67) | 1.29 (1.01, 1.66) | 1.3 (1.01, 1.67) | 1.3 (1.01, 1.67) | 1.3 (1.01, 1.67) | 1.3 (1.01, 1.67) |
| History of diabetes mellitus | 1.2 (0.95, 1.51) | 1.19 (0.94, 1.51) | 1.19 (0.94, 1.51) | 1.19 (0.94, 1.51) | 1.19 (0.94, 1.51) | 1.19 (0.94, 1.50) |
| Previous stroke | 1.31 (1.00, 1.71) | 1.31 (1.00, 1.70) | 1.3 (1.00, 1.70) | 1.3 (1.00, 1.70) | 1.31 (1.00, 1.71) | 1.3 (1.00, 1.70) |
| Previous warfarin use | 1.03 (0.84, 1.26) | 1.04 (0.85, 1.27) | 1.03 (0.84, 1.27) | 1.04 (0.85, 1.27) | 1.04 (0.85, 1.27) | 1.04 (0.85, 1.27) |
| Current amiodarone use | 0.98 (0.70, 1.36) | 0.97 (0.70, 1.35) | 0.96 (0.69, 1.34) | 0.96 (0.69, 1.34) | 0.97 (0.69, 1.34) | 0.96 (0.69, 1.33) |
| Current insulin use | 1.67 (1.13, 2.47) | 1.66 (1.12, 2.45) | 1.65 (1.11, 2.44) | 1.64 (1.11, 2.42) | 1.65 (1.12, 2.44) | 1.65 (1.11, 2.43) |
| Baseline use of β-blocker | 0.94 (0.77, 1.14) | 0.93 (0.77, 1.13) | 0.94 (0.77, 1.14) | 0.94 (0.77, 1.14) | 0.94 (0.77, 1.14) | 0.94 (0.77, 1.14) |
| Baseline use of aspirin | 1.24 (1.01, 1.52) | 1.24 (1.01, 1.51) | 1.24 (1.01, 1.51) | 1.24 (1.01, 1.52) | 1.24 (1.02, 1.52) | 1.24 (1.01, 1.51) |
| Baseline use of ace-inhibitor | 0.97 (0.80, 1.17) | 0.96 (0.79, 1.17) | 0.97 (0.80, 1.17) | 0.97 (0.80, 1.17) | 0.97 (0.80, 1.17) | 0.96 (0.79, 1.17) |
| Baseline use of statin | 1.09 (0.90, 1.33) | 1.09 (0.90, 1.33) | 1.09 (0.90, 1.33) | 1.09 (0.90, 1.33) | 1.09 (0.90, 1.33) | 1.1 (0.90, 1.33) |
| **Centre-level** |  |  |  |  |  |  |
| *Algorithm-consistent dosing (per 10%)* | 0.90 (0.69, 1.18) | 0.90 (0.82, 0.99) | 0.9 (0.83, 0.98) | 0.89 (0.81, 0.98) | 0.91 (0.82, 1.00) | 0.89 (0.81, 0.97) |
| Secondary/tertiary hospital | 0.82 (0.61, 1.10) | 0.80 (0.60, 1.07) | 0.83 (0.62, 1.11) | 0.82 (0.61, 1.09) | 0.82 (0.61, 1.09) | 0.81 (0.61, 1.08) |
| Anticoagulation clinic | 0.93 (0.71, 1.23) | 0.96 (0.73, 1.26) | 0.94 (0.72, 1.23) | 0.95 (0.72, 1.24) | 0.94 (0.72, 1.24) | 0.96 (0.73, 1.26) |
| **Country-level** |  |  |  |  |  |  |
| High income | 1.67 (1.13, 2.49) | 1.71 (1.17, 2.50) | 1.7 (1.16, 2.49) | 1.72 (1.19, 2.50) | 1.71 (1.17, 2.50) | 1.74 (1.19, 2.53) |
| Disability Adjusted Life Expectancy | 0.99 (0.94, 1.05) | 1.00 (0.95, 1.05) | 1 (0.95, 1.05) | 1 (0.95, 1.06) | 1 (0.95, 1.05) | 1 (0.95, 1.05) |
| Health System Performance Index | 0.20 (0.01, 2.76) | 0.14 (0.01, 1.77) | 0.14 (0.01, 1.81) | 0.11 (0.01, 1.37) | 0.13 (0.01, 1.65) | 0.13 (0.01, 1.57) |

### Association Between Centre-level Algorithm-Consistent Dosing and Adverse Events in Patients Randomized to Dabigatran in RE-LY

We re-fit the Cox proportional hazards model to assess the association between centre-level algorithm-consistent warfarin dosing in non-warfarin (dabigatran) patients in the trial used for evaluation (RE-LY) in order to establish the centre-level algorithm-consistent dosing is not a marker of generalized quality of care at a given centre.

**Table S3. Baseline patient characteristics of dabigatran vs warfarin groups used for time-to-event evaluation in RE-LY**

|  | **Warfarin**  **(n=5,730)** | **Dabigatran**  **(n = 6,794)** |
| --- | --- | --- |
| Age | 71.6 (8.5) | 72.2 (8.2) |
| Female (%) | 36.1 | 33.7 |
| Region (%) |  |  |
| East Asia | 12.1 | 3.8 |
| Eastern Europe | 15.5 | 11.1 |
| Latin America | 5.2 | 2.8 |
| North America | 36.4 | 54 |
| South Asia | 2.9 | 1.6 |
| Western Europe | 27.8 | 26.6 |
| Race (%) |  |  |
| Asian | 12.3 | 6.1 |
| Black | 1.1 | 1.2 |
| Other | 2.8 | 11.6 |
| White | 83.7 | 81.1 |
| Hispanic (%) | 16.5 | 2.8 |
| Weight (kg) | 81.8 (19.0) | 86.3 (20.1) |
| Systolic BP (mmHg) | 132.2 (16.2) | 130.0 (17.8) |
| BMI | 29.0 (5.7) | 29.5 (6.4) |
| Diabetes (%) | 39.5 | 25.6 |
| Hypertension (%) | 90.7 | 79.4 |
| CAD (%) | 23.4 | 34.1 |
| MI (%) | 18 | 20 |
| HF (%) | 7.2 | 31.2 |
| Stroke or TIA (%) | 8.8 | 20.2 |
| Paroxysmal AF | 62.2 | 31.9 |
| Ever smoked (%) | 51.8 | 58.7 |
| CHADS2 score | 17.1 | 2.2 (1.2) |
| CHADS2 = 1 (%) | 32.8 | 28.6 |
| CHADS2 = 2 (%) | 3.5 (0.9) | 34.4 |
| CHADS2 >= 3 (%) | 0 | 34.5 |
| Prior VKA use (%) | 13.2 | 100 |
| Aspirin (%) | 86.8 | 29.4 |
| Thienopyridines (%) | 62.4 | 4.6 |
| Beta blockers (%) | 36.4 | 67.1 |
| Calcium channel blockers (%) | 1.8 | 33.4 |
| Digoxin (%) | 65.4 | 30.5 |
| Proton pump inhibitors (%) | 27.3 | 16 |
| Creatinine | 38.6 | 1.4 (6.1) |
| **Adverse Events (% per patient-year)** |  |  |
| Death | 3.21 | 3.84 |
| Ischemic Stroke | 1.70 | 0.81 |
| Major Bleeding | 3.64 | 2.53 |
| Minor Bleeding | 21.46 | 7.50 |
| Hemorrhagic Stroke | 0.54 | 0.067 |
| Hospitalization | 9.92 | 16.27 |

*Continuous variables listed as mean (standard deviation). Categorical variables listed as %. BMI = body mass index, CABG = coronary artery bypass graft, CAD = coronary artery disease, MI = myocardial infarction, NSAID = non-steroidal anti-inflammatory, percutaneous coronary intervention, PPI = proton pump inhibitor, TIA = transient ischemic attack, VKA = vitamin K agonist.*

**Table S4.** **Multi-Level Multivariable Cox Proportional Hazard Model for Stroke, Systemic Embolism, or Major Hemorrhage for RE-LY patients on dabigatran**

| ***Characteristics*** | **Hazard Ratio (95% CI)** |
| --- | --- |
| **Patient-level** |  |
| Age (per year) | 1.05 (1.04, 1.06) |
| Weight (per kg) | 0.99 (0.99, 1.00) |
| Male | 1.14 (1.01, 1.29) |
| White | 0.79 (0.68, 0.92) |
| Current smoker | 1.42 (1.18, 1.71) |
| History of heart failure | 1.63 (1.45, 1.82) |
| History of hypertension | 1.17 (1.02, 1.34) |
| History of diabetes mellitus | 1.51 (1.34, 1.71) |
| Previous stroke | 1.37 (1.18, 1.58) |
| Previous warfarin use | 0.97 (0.86, 1.10) |
| Current amiodarone use | 1.08 (0.91, 1.30) |
| Current insulin use | 1.75 (1.45, 2.13) |
| **Centre-level** |  |
| *Algorithm-consistent dosing (per 10%)* | *0.96 (0.90, 1.02)* |
| Secondary/tertiary hospital | 1.26 (1.04, 1.52) |
| Anticoagulation clinic | 1.13 (0.95, 1.35) |
| **Country-level** |  |
| High income | 1.27 (0.98, 1.64) |
| Disability Adjusted Life Expectancy | 1.02 (0.98, 1.06) |
| Health System Performance Index | 0.21 (0.03, 1.33) |

### References

1. van Rossum, G. & Drake Jr, F. L. *Python reference manual*. (Centrum voor Wiskunde en Informatica Amsterdam, 1995).

2. Paszke, A. *et al.* PyTorch: An Imperative Style, High-Performance Deep Learning Library. (2019).

3. Moritz, P. *et al.* Ray: A Distributed Framework for Emerging AI Applications.

4. Mahmood, A. R., van Hasselt, H. & Sutton, R. S. Weighted importance sampling for off-policy learning with linear function approximation.

5. R Core Team. R: A Language and Environment for Statistical Computing. Preprint at https://www.R-project.org/ (2020).

6. Bates, D., Mächler, M., Bolker, B. & Walker, S. Fitting Linear Mixed-Effects Models Using lme4. *J Stat Softw* **67**, (2015).

7. Therneau, T. M. coxme: Mixed Effects Cox Models. Preprint at https://CRAN.R-project.org/package=coxme (2020).
